# Knowledge, attitudes, and practices toward cervical cancer prevention among women in Kampong Speu Province, Cambodia

**DOI:** 10.1186/s12885-018-4198-8

**Published:** 2018-03-15

**Authors:** Sothy Touch, Jin-Kyoung Oh

**Affiliations:** 1 Department of Cancer Control and Population Health, National Cancer Center Graduate School of Cancer Science and Policy, 323 Ilsan-ro, Ilsandong-gu, Goyang-si, Gyeonggi-do 410-769 Republic of Korea; 2 Cancer Risk Appraisal & Prevention Branch, National Cancer Center, 323 Ilsan-ro, Ilsandong-gu, Goyang-si, Gyeonggi-do 410-769 Republic of Korea

**Keywords:** Cervical cancer, Prevention, Screening, Human papillomavirus, Vaccination, Cambodia

## Abstract

**Background:**

There is little information concerning the preventive behaviors against cervical cancer among women in Cambodia, a country without organized cervical cancer screening programs and national human papillomavirus (HPV) vaccination policies. We aimed to examine the cervical cancer knowledge, attitudes, and practices as well as cervical cancer prevention methods among Cambodian women.

**Methods:**

A community-based cross-sectional survey on cervical cancer prevention was conducted. We conducted a face-to-face interview survey for women aged 20–69 years who lived in Kampong Speu Province. The data collection was conducted by a nurse and a trained health worker using a structured questionnaire from January 8 to February 19, 2016. The questionnaire comprised 46 questions on demographic and reproductive characteristics, knowledge of cervical cancer, related risk factors and preventive methods, and attitudes toward and practices of Pap test and HPV vaccination. A logistic regression analysis was used to evaluate the relationship between preventive behaviors against cervical cancer and related factors such as age, education, income, and knowledge of cervical cancer.

**Results:**

Among the 440 respondents, 74 and 34% of women had heard about cervical cancer and the Papanicolaou (Pap) Smear test, respectively, and 7% of women had ever been screened by a Pap test. The participants showed high willingness to undergo a Pap test (74%). Furthermore, 35% of women were aware that cervical cancer is preventable by vaccination and 62% of women were willing to get the HPV vaccine, but only 1% of women had been vaccinated against HPV. Women of a younger age (odds ratio: 76.7; 95% confidence interval: 19.2–306.5 among women aged 20–29 years compared to 60–69 years, P-for-trend< 0.0001) and those who were married (odds ratio: 2.8; 95% confidence interval: 1.3–6.3) were more likely to be willing to receive the vaccination.

**Conclusions:**

Women in the Kampong Speu province of Cambodia had a low awareness of cervical cancer screening and rarely practiced cervical cancer screening. However, the willingness to get Pap test and HPV vaccination is high.

## Background

Cervical cancer is one of the most common cancers in women worldwide and an important reproductive health problem in women. Approximately 85% of the global burden of cervical cancer occurs in less-developed regions, where it accounts for almost 12% of all cancers in women [1].

The prevalence of human papillomavirus (HPV), an important cause of cervical cancer, is higher in less-developed countries than in more-developed regions [2, 3]. The majority of deaths due to cervical cancer occur in women who were never screened or treated as well as those who had an early sexual debut, a history of multiple sexual partners, and a high number of live births [4]. Strong evidence shows that the progression of cervical cancer into its later stages can be prevented through screening and treatment of premalignant lesions. Thus, in developed countries, the incidence of cervical cancer has been controlled due to effective screening programs, especially the systematic use of the Papanicolaou (Pap) smear test for identifying premalignant changes in the cervix [5]; however, in many developing countries, screening services are lacking or are poorly accessible for the majority of the population [6]. In Cambodia, a country with medium human development [7], cervical cancer is the most-common cause of cancer in women. There is no data registry for cancer in Cambodia, the cancer incidence and mortality rates are estimated from those of neighboring countries or registries in the same area (i.e., Vietnam and Thailand) [8]. In 2012, the age-standardized incidence and mortality rate of cervical cancer were 23.8 and 13.4, respectively, rates that are 3 times higher than those in Singapore [9]. The majority of women affected with cancer in Cambodia present to the clinic/hospital with an incurable advanced clinical stage of disease, which often has a very poor prognosis, eventually resulting in death [10]. In Cambodia, there are no quality data on the cancer burden and no systematic cervical cancer-screening programs and national or governmental HPV vaccination policies [11]. Furthermore, there is little information available on the preventive behaviors against cervical cancer among women in Cambodia.

Therefore, this study aimed to investigate the knowledge, attitudes, and practices (KAP) toward cervical cancer screening and HPV vaccination by conducting a KAP survey in a rural area in Cambodia.

## Methods

### Study participants

A community-based cross-sectional KAP survey on cervical cancer prevention was conducted for women aged 20–69 years in Kampong Speu Province, Cambodia, between January 8 and February 19, 2016. Kampong Speu is a rural area located in the southwestern part of Cambodia with 8 districts: Aural, Baset, Chbar Mon, Kong Pisei, Phnom Srouch, Samraong Tong, Thpong, and Udong. Most people living in Kampong Speu belong to the low- and middle-income groups, and the main economic activities in the province are agriculture and industry. For each district, we aimed to interview an equal number of participants in each age category (i.e., 20–29, 30–39, 40–49, 50–59, and 60–69 years). In each district, streets were chosen at random and houses were visited sequentially until the predetermined number of surveys was completed. A face-to-face interview survey of female household members was conducted by trained interviewers using a structured questionnaire. We developed the KAP questionnaire to use in this study. An English version of the questionnaire was developed and it was translated into Khmer version. The English and Khmer versions of the questionnaire were pre-tested in a small group of women before survey to validate and modify the questionnaire. Women who had a hysterectomy or a history of cancer as well as women who were not mentally fit to answer the questions were excluded from the survey. After excluding 5 women who were not eligible for the survey among 445 in total contacted women, 440 women completed the interview. All study participants provided written informed consent before the survey. This study was approved by the National Ethics Committee for Health Research in Cambodia.

### Measures

The questionnaire comprised 46 questions on demographic and reproductive characteristics, knowledge of cervical cancer, related risk factors and preventive methods, and attitudes toward and practices of Pap test and HPV vaccination. Demographic characteristics included age, education level, occupation, family monthly income, and marital status. Reproductive characteristics included number of children, family history of cervical cancer, history of sexually transmitted diseases and contraceptive use, number of sexual partners, and smoking and alcohol habits. In addition, knowledge of cervical cancer and related risk factors, Pap test, HPV vaccination, source of information, and health-seeking behavior were also measured. To understand women’s attitudes and practices, questions focusing on 5 concepts were adapted from the Health Belief Model: perceived severity, perceived susceptibility, perceived benefits, perceived barriers, and cues to action.

For data collection through the survey, most of the questions were close-ended, i.e., the responses were limited to “Yes,” “No,” and “I do not know,” and some questions had multiple-choice responses. To obtain additional opinions, open-ended questions were also used. The responses to the open-ended questions were categorized into the most relevant pre-existing choices.

### Statistical analysis

Categorical variables are presented as numbers or percentages. Differences in distribution were identified using the Pearson chi-square test. A logistic regression analysis was used to evaluate the relationship between preventive behaviors (i.e., Pap test or HPV vaccination) against cervical cancer and related factors such as age, education, income, and knowledge of cervical cancer. Odds ratios (ORs) and 95% confidence intervals (CIs) were also calculated. All analyses were carried out using SAS (version 9.3; SAS Institute, Cary, NC).

## Results

Table 1 shows the socio-demographic and reproductive characteristics of the respondents. Among the respondents, most women had a low education level (75% with no education or primary school education), worked as a farmer or in fisheries (41%), and earned a low or modest level of income (93% with monthly family income under 375 US dollar). Most women were married (81%) with 3 or more children (67%), were non-smokers (99%), were non-alcohol drinkers (79%), and had 1 or 2 sexual partners (94%).

**Table 1:** Socio-demographic and reproductive characteristics of women included in the study (*N* = 440)

Variables	Number	Percent
Age (in year)		
20–29	88	20.0
30–39	88	20.0
40–49	88	20.0
50–59	88	20.0
60–69	88	20.0
Education		
No school	125	28.4
Primary school	205	46.5
≥ Secondary school	110	25.0
Occupation		
Self-employed	60	13.6
Factory worker	62	14.0
Housewife/unemployed	114	25.9
Farmer/Fishery	180	40.9
Others^a^	24	5.4
Family income, monthly		
Low (US$ 0–124)	192	43.6
Middle (US$ (125–374)	216	49.0
High (≥US$ 375)	32	7.3
Marital status		
Married	356	80.9
Single^b^	84.0	19.0
Number of children		
No children	19	4.6
1 or 2 children	119	28.8
3 or 4 children	275	66.5
Mean (SD)	2.6 ± 0.5	
Family history of cervical cancer		
No	178	40.4
Yes	9	2.0
Do not know	253	57.5
History of sexually transmitted diseases		
No	355	80.6
Yes	14	3.1
Do not know	71	16.1
Contraceptive use		
No	292	66.3
Yes	148	33.6
Smoking habit		
Never smoked	437	99.3
Current/former smoker	3	0.6
Alcohol drinking		
No	345	78.5
Sometimes	94	21.4
Number of lifetime sexual partner		
None	26	5.9
1 or 2	403	91.6
≥ 3	11	2.5

^a^student, labor, school teacher, employee of private company, head of village, accountant and midwifery

^b^unmarried, divorced, separated and widowed

Table 2 shows women’s KAP toward cervical cancer and the Pap test. Most women had ever heard about cervical cancer (74%), but a limited number of women had ever heard about the Pap test (34%). Many women (46%) were aware that having multiple sex partners is a risk factor for cervical cancer, but only 2% of women were aware that HPV infection too was a risk factor for cervical cancer. Many women (85%) were aware that cervical cancer is a serious disease, but only 7% of women ever underwent a Pap test, as they had no symptom and believed that the Pap test was not necessary. Further, 74.3% of women were willing to undergo a Pap test. After adjustment, our results showed that women of younger age (P for trend < 0.001) and with knowledge of the Pap test (OR = 1.8; 95% CI: 1.0–3.1) were more likely to be willing to undergo a Pap test (Table 4).

**Table 2:** Knowledge, attitude, and practice toward cervical cancer and Papanicolaou test in women included in the study (*N* = 440)

Variables	Number	Percent
Had ever heard about cervical cancer		
No	114	25.9
Yes	326	74.0
Had ever heard about the Pap test		
No	288	65.6
Yes	151	34.4
Information source		
From a medical staffs or a hospital	15	9.8
Radio, TV newspaper	60	39.4
Others^a^	77	50.6
Cervical cancer can be detected early by screening		
No	256	58.2
Yes	184	41.8
The most important risk factor of cervical cancer		
Having many sexual partner	204	46.3
Having many child birth	57	12.9
Smoking	31	7.0
Old age	10	2.2
Human papilloma virus	8	1.8
Alcohol drinking	7	1.5
Do not know	123	27.9
The optimal frequency of the Pap test		
Every 3 year	89	20.2
When symptom appears	70	15.9
Every 1 or 2 years	67	15.2
From age 30 with 3 to 5 years interval	38	8.6
Every 6 months	15	3.4
Once in a lifetime at any age	6	1.3
Don’t know	155	35.2
Cervical cancer is a fatal disease		
No	66	15.0
Serious but curable disease	143	32.5
Very fatal disease	231	52.5
Health seeking behavior when symptom appears		
Go to health center	236	53.6
Consult with doctor immediately	93	21.1
Visit Reproductive Health Association of Cambodia	58	13.1
Got to a traditional healer	23	5.2
Others^b^	30	6.8
Had ever had the Pap test		
No	409	92.9
Yes	31	7.0

^a^family member, relative, friend, school, NGO, missionary, lecture and health magazine

^b^Oriental medicine, village nurse

Table 3 shows women’s KAP towards HPV infection and vaccination. Few women (8.6%) were aware that HPV infection is transmitted by sexual contact, and 35.2% of women were aware that cervical cancer is preventable by vaccination. Only 6 women (1.3%) received an HPV vaccination and 62% of women were willing to receive vaccination for themselves as well as their daughters. The high cost of vaccination and lack of knowledge about the vaccine were the most important barriers to HPV vaccination. Women of a younger age and those who were married were more likely to be willing to receive the vaccination (Table 4).

**Table 3:** Knowledge, attitude, and practice toward human papillomavirus and vaccination in women included in the study (*N* = 440)

Variables	Number	Percent
HPV infection is transmitted by sexual contact		
No	402	91.3
Yes	38	8.6
Cervical cancer is preventable by vaccination		
No	285	64.7
Yes	155	35.2
Had done the HPV vaccination		
No	434	98.6
Yes	6	1.3
Willingness to be vaccinated against HPV, for free		
No	89	20.2
Yes	273	62.0
Do not know	78	17.7
Willingness to be vaccinated against HPV, by your payment		
No	164	37.2
Yes	157	35.6
Do not know	119	27.0
Willingness to pay for the HPV vaccine, per shot		
Mean (USD)	20.5 ± 8.1	
Willingness to vaccinate your daughter against HPV		
No	21	4.7
Yes	273	62.0
Do not know	146	33.1
The biggest reason for not having the HPV vaccination		
High cost	93	32.7
Lack of knowledge about HPV	71	25.0
Don’t know where to get HPV vaccine	13	4.5
Don’t trust vaccine safety	15	5.2
No risk as not exposed to sexual contact	10	3.5
Others^a^	82	28.8
The best time to be vaccinated against HPV		
Before sexual contact	182	41.3
After sexual contact or child birth	44	10.0
After marriage or at any time	38	8.6
Do not know	176	40.0

^a^Too old to be vaccinated, healthy, afraid of injection, husband not allows injection

**Table 4:** Odds ratios and 95% confidence intervals of willingness to undergo a Papanicolaou test and human papillomavirus vaccination according to selected variables among women included in the study (*N* = 400)

Selected Variables	Total†	Willingness to do Pap-test	Crude OR (95%CI)	Adjusted ORª(95%CI)	Willingness to be vaccinated against HPV	Crude OR (95% CI)	Adjusted ORª (95% CI)
Age (in years)							
60–69	88 (20.0)	47 (53.4)	Ref	Ref	19 (21.6)	Ref	Ref
20–29	88 (20.0)	73 (82.9)	4.2 (2.1–8.5)	3.2 (1.0–10.1)	81 (92.0)	42.0 (16.6–105.8)	76.7 (19.2–306.5)
30–39	88 (20.0)	76 (86.3)	5.5 (2.6–11.5)	4.4 (1.8–11.0)	70 (79.5)	14.1 (6.8–29.1)	24.8 (7.8–79.0)
40–49	88 (20.0)	71 (80.7)	3.6 (1.8–7.1)	3.5 (1.6–7.5)	59 (67.0)	7.3 (3.7–14.5)	15.9 (5.1–49.5)
50–59	88 (20.0)	60 (68.2)	1.8 (1.0–3.4)	1.8 (0.9–3.5)	45 (51.1)	3.8 (1.9–7.3)	6.8 (2.2–20.9)
				*p*-trend <.0001			*P* –trend<.0001
Education							
No school	125 (28.4)	80 (64.0)	Ref	Ref	59 (47.2)	Ref	Ref
Primary school	205 (46.6)	158 (77.1)	1.8 (1.1–3.0)	1.4 (0.8–2.4)	127 (61.9)	1.8 (1.1–2.8)	1.1 (0.6–2.3)
≥ Secondary school	110 (25)	89 (80.9)	2.3 (1.3–4.3)	1.0 (0.4–2.4)	88 (80.0)	4.4 (2.4–8.0)	0.9 (0.3–2.5)
				*p*-trend = 0.331			*P* -trend = 0.199
Occupation							
Housewife/unemployed	114 (25.9)	78 (68.4)	Ref	Ref	58 (50.9)	Ref	Ref
Self-employed	60 (13.6)	47 (78.3)	1.7 (0.8–3.4)	0.9 (0.3–2.4)	43 (71.7)	2.4 (1.2–4.8)	1.5 (0.5–4.3)
Factory worker	62 (14.1)	52 (83.9)	2.4 (1.1–5.3)	0.9 (0.3–2.7)	52 (83.9)	5.0 (2.3–10.8)	1.6 (0.5–4.8)
Farmer/Fishery	180 (40.9)	133 (73.9)	1.3 (0.7–2.1)	1.2 (0.7–2.2)	101 (56.1)	1.2 (0.8–1.9)	1.2 (0.5–2.5)
Other^b^	24 (5.4)	17 (70.8)	1.1(0.4–3.0)	0.5 (0.0–2.7)	20 (83.3)	4.8 (1.5–15.0)	2.9 (0.6–14.1)
Family Income/monthly							
Low (US$ 0–124)	192 (43.6)	127 (66.1)	Ref	Ref	92 (47.9)	Ref	Ref
Middle (US$ (125–374)	216 (49.1)	173 (80.1)	2.0 (1.3–3.2)	1.1 (0.6–2.0)	160 (74.1)	3.1 (2.0–4.7)	1.0 (0.5–2.1)
High (≥US$ 375)	32 (7.3)	27 (84.2)	2.7 (1.0–7.5)	1.3 (0.4–4.9)	22 (68.7)	2.3 (1.0–5.3)	0.5 (0.1–1.9)
				*p*-trend = 0.610			*P* -trend = 0.714
Marital Status							
Single	84 (19.1)	50 (59.5)	Ref	Ref	41 (48.8)	Ref	Ref
Married	356 (80.9)	277 (77.8)	2.3 (1.4–3.9)	1.7 (0.9–3.3)	233 (65.4)	1.9 (1.2–3.2)	2.8 (1.3–6.3)
Number of Children							
No children	19 (4.6)	13 (68.4)	Ref	Ref	14 (73.7)	Ref	
1 or 2 children	119 (28.8)	104 (87.4)	3.2 (1.0–9.6)	2.3 (0.7–7.6)	96 (80.7)	1.4 (0.4–4.5)	–
3 or 4 children	275 (66.6)	194 (70.5)	1.1 (0.4–3.0)	1.1 (0.3–3.7)	141 (51.3)	0.3 (0.1–1.0)	–
Had ever heard about Cervical Cancer							
No	114 (25.9)	80 (70.2)	Ref	–	114 (25.9)	Ref	Ref
Yes	326 (74.1)	247 (75.5)	1.3 (0.8–2.1)	–	326 (74.1)	2.1 (1.4–3.3)	2.0 (1.0–4.2)
Had ever heard about Pap test							
No	288 (65.6)	201 (69.8)	Ref	Ref			
Yes	151 (34.4)	126 (83.4)	2.1 (1.3–3.5)	1.8 (1.1–3.3)			
Cervical cancer is preventable							
No	123 (28.0)	99 (80.5)	Ref	–	123 (27.9)	Ref	Ref
Yes	317 (72.1)	228 (71.9)	0.6 (0.3–1.0)	–	317 (72.0)	0.5 (0.3–0.8)	0.7 (0.3–1.5)
Cervical cancer is a fatal disease							
No	66 (15.0)	45 (68.2)	Ref	–	66 (15.0)	Ref	Ref
Serious but curable disease	143 (32.5)	108 (75.5)	1.4 (0.7–2.7)	–	143 (32.5)	2.6 (1.4–4.7)	1.7 (0.6–4.7)
Very fatal disease	231 (52.5)	174 (75.3)	1.4 (0.7–2.5)	–	231(52.5)	1.8 (1.0–3.1)	1.7 (0.7–4.3)
Had ever heard about HPV vaccine							
No					285 (64.7)	Ref	Ref
Yes					155 (35.2)	2.6 (1.7–4.0)	1.2 (0.6–2.4)
Cervical cancer can be detected early by screening							
No	40 (9.1)	27 (67.5)	Ref	–			
Yes	184 (41.8)	146 (79.3)	1.8 (0.8–3.9)	–			
Do not know	216 (49.1)	154 (71.3)	1.1 (0.5–2.4)	–			
Number of sexual partner							
None	26 (5.9)	17 (65.4)	Ref	–			
1 to more than 2	414 (94.1)	310 (74.9)	1.5 (0.6–3.6)	–			

^a^Adjusted for significant variables in the unadjusted model

^b^student, labor, school teacher, employee of private company, head of village, accountant and midwifery

Note: Sample size in each variable may not equal due to missing value

## Discussion

In Cambodia, cervical cancer is the most-common cause of cancer in women. There is no data registry for cancer in Cambodia, the cancer incidence and mortality rates are estimated from those of neighboring countries or registries in the same area (i.e., Vietnam and Thailand). The GLOBOCAN, a major source of cancer incidence and mortality worldwide provided by the International Agency for Research on Cancer and World Health Organization, estimated the incidence rate in Cambodia as the mean average of the incidence rates from: 1) Sex- and age-specific incidence in all sites from Viet Nam, Ho Chi Min City (2006–2010) partitioned by site and age using proportions from Phnom Penh Cancer Registry (2001–2003); 2) Simple mean of the rates from Thailand, Ubon Ratchathani (2004–2006) and Rayong (2004–2006) cancer registries [8]. The mortality was estimated from national cancer incidence estimates using modeled survival. In 2012, the estimated age-standardized incidence and mortality rate of cervical cancer in Cambodia were 23.8 and 13.4, respectively [9].

In many developing countries, women’s knowledge of cervical cancer and preventive measures is limited. In addition, the screening rate of cervical cancer is low in low-income countries. For example, studies have reported that only 13–29% of women in North Korea [12] and 28% in Gabon [13] are aware of cervical cancer screening, and 15% of women in India [14], 26% in Malaysia [15], 32% in Nepal [16], and 36% in Thailand [17] are aware of the HPV vaccine.

In this study, 74% of study women living in Kampong Speu, Cambodia, had ever heard about cervical cancer, 34% of women had ever heard about the Pap test, and only 7% of women ever underwent a Pap test. These findings show that the level of knowledge about cervical cancer screening remains low among this population, which can explain why most patients with cervical cancer present to the clinic late with an advanced stage of disease. Education the public about the cervical cancer is low. Cultural norms often prevent women from speaking up or seeking treatment if they do not have any symptoms. Women get a screening at local health centers, but must be referred to a district hospital for treatment. Both primary national hospitals offering oncology treatment are located only in capital, Phnom Penh [10].

In this study, we also found that 39% of respondents listed the city media (radio/television), followed by medical staffs/hospital (10%), as their source of information of the Pap test. In addition, a majority of the participants reported having either a radio or television in their homes, which shows that the media plays an important role in disseminating health educational information. Therefore, there is need for a health-education program about cervical cancer that incorporates the media through diverse channels; such a program could be very impactful. Furthermore, given that the second most-common source was hospitals/medical staff, access to healthcare should be improved in the future. According to a WHO’s report, the availability of public health facilities has increased in Cambodia. There have been significant increases in the proportion of women attending antenatal care visits, and delivering at health facilities [18]. Improved availability of and demand for skilled maternity care can be an opportunity to provide information on Pap test.

With regard to risk factors, 47 and 2% of women reported multiple sexual partners and HPV infection, respectively, as the most important risk factors of cervical cancer. According to a systematic review, which included 39 studies across 11 countries, overall knowledge of the general public about HPV infection is poor, and the findings support our results [19].

In general, the poor uptake of the Pap test could be explained by the fact that people worldwide do not usually undergo health checkups until they experience health problems; therefore, the absence of systematic and active promotion of a screening program in the country may contribute to low utilization of the Pap test. Furthermore, in Cambodia, there is no organized cervical cancer-screening program. Although HPV vaccination has been introduced into two provinces – Svay Rieng and Siem Reap - as part of the demonstration project very recently, they have not been implemented in the national immunization program [11]. In addition, healthcare resources for screening, evaluating, and treating abnormal cases (including trained health personnel, hospitals, and clinics for quality cytological testing) are limited in Cambodia. Nevertheless, this study shows that the women of Cambodia were highly willing to undergo the Pap test (74% of the participants). We did not provide an active education in Pap test during the survey. However, the respondents came to know about Pap test through the survey (informed consents and introduction to the study purpose, etc). The study participants had little chance to meet health workers so they gladly consulted the interviewers who are trained nurses about their health issues. Although the knowledge on cervical cancer and preventive measures were low, their willingness to prevent disease was so high. Therefore, interventions should be targeted toward improving access to screening for cervical cancer. Further, 52% of women were not aware that the Pap test should be performed regularly and believed that it is needed only when a symptom appears or once in a lifetime at any age. This misconception may help explain the low uptake of the Pap test (7%) among women in this study, and it is critical to raise awareness regarding the importance of regular screening in this population.

HPV vaccination can be an effective method to prevent cervical cancer, especially in a country with limited healthcare resources for screening and treatment. In this study, 35% of women were aware that cervical cancer is preventable by vaccination and 62% of women were willing to receive the HPV vaccine, but only 1% of women had been vaccinated against HPV. The willingness to vaccinate HPV vaccine to their girls was also high (62%). However, high cost and lack of knowledge of HPV vaccination were the biggest barriers to vaccination in this study. Therefore, in order to increase the vaccine coverage in Cambodia, it is important to increase awareness of the HPV vaccine and decrease the cost of the vaccine to make it affordable.

According to the United Nations Population Fund (UNFPA), HPV vaccine was introduced into the routine immunization system in Cambodia since 2017, starting with the two provinces – Svay Rieng and Siem Reap as part of the demonstration project. A total of 4850 girls aged 9-year old residing in Svay Rieng province will receive 2 doses of the vaccine free of charge from health centers and through outreach activities to schools and health centers. The first dose was offered in January while the second dose took place in July 2017. GAVI, the Vaccine Alliance has provided financial support to purchase the HPV vaccine while WHO, UNICEF, UNFPA and other stakeholders have actively advocated for its inclusion into the national vaccination program [19].

Despite our important findings, this study has a several potential limitations. First, the sample size was modest (*N* = 440), and the results from this study cannot be generalized to all Cambodian women. According to census data in 2008, the actual proportion of women in the study area is high in young age group (20–29 years old) and decreases followed by age. However, in considering with statistical power in old age groups which are more affected age group by cervical cancer, same number of study participants (i.e. oversampling in old age groups) was recruited in each age category. Second, some of the questions might be leading. For example, “Do you think cervical cancer can be detected early by screening?” may lead more positive answer than a more neutral question such as “Can cervical cancer be detected through screening?” The questionnaire was asked in Cambodian language, Khmer, and the actual meaning and tone might vary by interviewer. Third, some of the confidence intervals in the results are very wide because of the small sample size. When interpret the results with large confidence interval, p-for-trend should also be considered. Lastly, some respondents may not be able to clearly distinguish between gynecological examination and a Pap test, and the frequency of the Pap test may have been overestimated. Thus, large-scale studies among Cambodian women regarding KAP toward cervical cancer prevention are needed in the future.

Nonetheless, this study has many strengths. For example, this is the first study conducted in the community to investigate women’s KAP toward cervical cancer prevention in Cambodia. In addition, this study also had a very high response rate (100%). This is most likely because the women contacted had an opportunity to receive advice about their health concerns from trained health personnel, and the study was introduced by the head of village using an official document from the Cambodia National Ethics Committee.

## Conclusions

In conclusion, this study showed that women in the Kampong Speu province of Cambodia had a low awareness of cervical cancer screening and rarely practiced cervical cancer screening. However, the willingness to get Pap test and HPV vaccination is high. Developing strategies and implementing effective programs for cervical cancer prevention in the resource-constrained setting are needed.

## Abbreviations

CI: confidence interval
HPV: human papillomavirus
KAP: knowledge, attitudes, and practices
OR: Odds ratio
Pap: Papanicolaou
